# DEMENTIA PROGRAMS FOR PERSONS WITH INTELLECTUAL OR DEVELOPMENTAL DISABILITIES (IDD) AND THEIR CARE PARTNERS

**DOI:** 10.1093/geroni/igae098.1687

**Published:** 2024-12-31

**Authors:** Kelli Barton

**Affiliations:** University of Missouri-Kansas City Institute for Human Development, Missouri, United States

## Abstract

There are limited evidence-based dementia programs for individuals with intellectual or developmental disabilities (IDD) and their care partners. Alzheimer’s Disease Programs Initiative grants through the Administration for Community Living provide an opportunity to expand evidence-based dementia programs to serve the IDD population. This presentation will discuss outcomes and lessons learned from the implementation of two dementia programs, Reducing Disability in Alzheimer’s Disease (RDAD) and Care Ecosystem, with the IDD population and their care partners through three funded projects. We employed a mixed-method approach to explore program impact and identify lessons learned that can inform dementia programming for the IDD population. Feedback from semi-structured partner interviews and regular meetings revealed several common themes. Partners identified the opportunity to provide dementia and IDD education to staff and to the community as a significant benefit and as crucial to establishing a foundation for program implementation. Further, while many care partners were formal caregivers, whose relationship and experience with the person with dementia typically differ from informal caregivers, results from the RDAD implementation showed promising trends in outcomes for both individuals and care partners. For example, behavioral symptom-related severity among individuals with IDD and behavioral symptom-related distress among care partners both decreased (p=0.07 and p=0.14, respectively), as measured by the Neuropsychiatric Inventory Questionnaire (NPI-Q). Evidence-based dementia programs have much to offer the IDD population and their care partners; however, protocol modifications are needed to ensure physical and cognitive accessibility for individuals with IDD and to meet the needs of informal caregivers.

